# Coronary artery calcium score on standard of care oncologic CT scans for the prediction of adverse cardiovascular events in patients with non-small cell lung cancer treated with concurrent chemoradiotherapy

**DOI:** 10.3389/fcvm.2022.1071701

**Published:** 2022-12-02

**Authors:** Efstratios Koutroumpakis, Ting Xu, Juan Lopez-Mattei, Tinsu Pan, Yang Lu, Jorge A. Irizarry-Caro, Radhe Mohan, Xiaodong Zhang, Qing H. Meng, Ruitao Lin, Tianlin Xu, Anita Deswal, Zhongxing Liao

**Affiliations:** ^1^Division of Internal Medicine, Department of Cardiology, The University of Texas MD Anderson Cancer Center, Houston, TX, United States; ^2^Division of Radiation Oncology, Department of Radiation Oncology, The University of Texas MD Anderson Cancer Center, Houston, TX, United States; ^3^Lee Health Heart and Vascular Institute, Lee Health System, Fort Myers, FL, United States; ^4^Division of Diagnostic Imaging, Department of Imaging Physics, The University of Texas MD Anderson Cancer Center, Houston, TX, United States; ^5^Division of Diagnostic Imaging, Department of Nuclear Medicine, The University of Texas MD Anderson Cancer Center, Houston, TX, United States; ^6^Department of Internal Medicine, The University of Texas Health Science Center at Houston, Houston, TX, United States; ^7^Division of Radiation Oncology, Department of Radiation Physics, The University of Texas MD Anderson Cancer Center, Houston, TX, United States; ^8^Division of Pathology/Lab Medicine, Department of Laboratory Medicine, The University of Texas MD Anderson Cancer Center, Houston, TX, United States; ^9^Division of Basic Science Research, Department of Biostatistics, The University of Texas MD Anderson Cancer Center, Houston, TX, United States

**Keywords:** coronary artery calcification (CAC), calcium score, cardiooncology, non-small cell lung carcinoma, adverse cardiovascular events, overall survival, mortality

## Abstract

**Introduction:**

Chemoradiotherapy (CRT) has been associated with increased incidence of cardiovascular (CV) adverse events (CVAE). Coronary artery calcium scoring (CAC) has shown to predict coronary events beyond the traditional CV risk factors. This study examines whether CAC, measured on standard of care, non-contrast chest CT (NCCT) imaging, predicts the development of CVAE in patients with non-small cell lung cancer (NSCLC) treated with CRT.

**Methods:**

Patients with NSCLC treated with CRT at MD Anderson Cancer Center from 7/2009 until 4/2014 and who had at least one NCCT scan within 6 months from their first CRT were identified. CAC scoring was performed on NCCT scans by an expert cardiologist and a cardiac radiologist following the 2016 SCCT/STR guidelines. CVAE were graded based on the most recent Common Terminology Criteria for Adverse Events (CTCAE) version 5.0. CVAE were also grouped into (i) coronary/vascular events, (ii) arrhythmias, or (iii) heart failure. All CVAE were adjudicated by a board-certified cardiologist.

**Results:**

Out of a total of 193 patients, 45% were female and 91% Caucasian. Mean age was 64 ± 9 years and mean BMI 28 ± 6 kg/m^2^. Of 193 patients, 74% had CAC >0 Agatston units (AU), 49% CAC ≥100 AU and 36% CAC ≥300 AU. Twenty-nine patients (15%) developed a grade ≥2 CVAE during a median follow-up of 24.3 months (IQR: 10.9–51.7). Of those, 11 (38%) were coronary/vascular events. In the multivariate cox regression analysis, controlling for mean heart dose and pre-existing CV disease, higher CAC score was independently associated with development of a grade ≥2 CVAE [HR: 1.04 (per 100 AU), 95% CI: 1.01–1.08, *p* = 0.022] and with worse overall survival (OS; CAC ≥100 vs. <100 AU, HR: 1.64, 95% CI: 1.11–2.44, *p* = 0.013). In a sub-analysis evaluating the type of the CVAE, it was the coronary/vascular events that were significantly associated with higher baseline CAC (median: 676 AU vs. 73 AU, *p* = 0.035).

**Discussion:**

Cardiovascular adverse events are frequent in patients with NSCLC treated with CRT. CAC calculated on “standard of care” NCCT can predict the development of CVAEs and specifically coronary/vascular events, as well as OS, independently from other traditional risk factors and radiation mean heart dose.

**Clinical trial registration:**

[https://clinicaltrials.gov/ct2/show/NCT00915005], identifier [NCT00915005].

## Introduction

Radiation therapy (RT) is an integral part of the treatment for non-small cell lung carcinoma (NSCLC). In a recent study including more than 288,000 subjects with NSCLC, RT was used in one third (1). Despite its significant benefits in the treatment of NSCLC, RT has been associated with increased incidence of cardiovascular (CV) adverse events (CVAEs) including coronary events, arrhythmias, and heart failure (2, 3). In a recent meta-analysis of four prospective radiation therapy trials that included patients with NSCLC stage II–III treated between 2004 and 2013, approximately 1 out of 10 participants (11%) developed a significant CVAE within 2 years from RT initiation (3). Higher mean heart dose (MHD) and pre-existing cardiovascular disease (CVD) have been associated with higher incidence of CVAEs (2–4). More recent studies suggest that critical cardiac substructure dose, such as left ventricular or left anterior descending artery dose as opposed to left circumflex or right coronary artery dose, may predict the development of future CVAEs more accurately than MHD (5, 6). Contemporary RT modalities, such as 3-dimensional conformal RT (3DCRT), intensity modulated RT (IMRT), volumetric modulated arc radiation (VMAT), RT with respiratory gating and proton beam therapy, have managed to lower the MHD, and cardiac substructure dose (7, 8). Despite improvement in radiation modalities and cardiovascular shielding techniques, radiation induced CVD remains a common cause of morbidity and mortality among NSCLC survivors (9, 10). Early diagnosis of subclinical atherosclerosis, such as identification of coronary calcification, has the potential to better risk stratify patients with NSCLC and potentially guide preventive pharmacotherapy with statins, which has shown some promise in mitigating radiation induced atherosclerotic events (11).

Coronary artery calcium scoring (CAC) on CT imaging, has emerged as a widely available, cost-effective and reproducible means of detecting pre-clinical coronary atherosclerosis (12). It improves prediction of major CV outcomes beyond assessment of traditional CV risk factors, and it is especially useful in asymptomatic individuals for planning primary prevention interventions such as initiation of statins (12, 13). Although CAC was initially developed based on ECG-gated, non-contrast, prospective cardiac CT imaging (14), CAC on standard, non-gated, non-contrast CT (NCCT) imaging has been shown to correlate well with the traditional CAC and has been proposed as a cost-effective method of screening patients with lung cancer prior to RT (15, 16). Furthermore, in a recent study from our institution, coronary artery radiation exposure was associated with subsequent increase in CAC in patients with cancer (17). In this study, we investigate the role of CAC measured on standard of care, oncologic NCCT scans in predicting CVAEs in patients with NSCLC treated with concurrent chemoradiation including different modalities of RT.

## Materials and methods

### Study population

This is a sub study of a prior randomized clinical trial (clinical trial # NCT00915005) (18). We screened 238 consecutive patients with NSCLC that were part of the trial and started receiving either photo-radiation or proton therapy from 7/1/2009 to 04/30/2014 at MD Anderson Cancer Center. All patients also received concurrent chemotherapy. Patients who had at least one NCCT within 6 months from the first day of their RT were included. NCCT scans were either part of the patients’ PET-Scan protocol or standalone NCCT scans for lung cancer staging and they were used for quantifying CAC score. CT scans performed for RT planning were not included due to lower spatial resolution and limited image quality to evaluate heart substructures. After applying our inclusion criteria, the study population of this study was limited to 193 patients.

### Data collection

The patient demographic and clinical parameters were collected prospectively as part of the randomized clinical trial (clinical trial # NCT00915005) (18). These variables included age, sex, race, body mass index (BMI), pre-existing CV disease, cancer characteristics such as histology, staging, gross tumor volume, and information regarding RT including technique, mean heart dose, and total radiation dose. Pre-existing CV disease included coronary artery disease, carotid artery disease, peripheral artery disease, aortic aneurysm, sustained atrial or ventricular arrhythmias, heart failure or cardiomyopathy, and pericardial disease.

### Coronary artery calcification scoring

Coronary artery calcification scoring was performed following the 2016 SCCT/STR guidelines for CAC scoring on NCCT scans (19). Every NCCT scan prior to RT was uploaded into a workstation (Syngo.*Via*, Siemens Healthcare, Malvern, PA, USA). CAC score was quantified in each study using the CT Ca Scoring application from the CT Cardiac package of Syngo.*Via* (Siemens Healthcare, Malvern, PA, USA). Each study was processed by a board-certified cardiologist, level 3 COCATs trained in Cardiac CT and cross verified by a cardiac radiologist. A meticulous assessment of all coronary territories was performed for CAC assessment, making sure that mitral annular calcification, valvular calcification and cardiac implants did not interfere with the CAC calculation. Total and individual coronary artery CAC scores were collected. Presence of coronary stents was noted.

### Outcomes

Cardiovascular adverse events were collected by a retrospective patient chart review. CVAEs were graded based on severity using the most recent Common Terminology Criteria for Adverse Events (CTCAE) version 5.0 published by the National Cancer Institute (NCI) of the National Institutes of Health (NIH) (20). CVAEs were also grouped into the following categories: (i) coronary/vascular events, (ii) sustained supraventricular or ventricular arrhythmias, and (iii) heart failure. All cardiac events were adjudicated by a board-certified cardiologist. All-cause mortality/overall survival (OS) was also collected.

### Statistical analysis

Continuous variables are presented as mean values +/− standard deviation (SD) and were compared using the student’s *t*-test. Categorical variables are presented as percentages and were compared using the χ2 test. Time to an adverse event was computed from the start of RT to the date of the first documented CVAE. Patients who did not develop a CVAE were censored at the time of the last follow-up or death. Cumulative incidences of CVAE and OS were estimated by the Kaplan–Meier method and compared with log-rank tests for differences between groups. CAC scores were compared in CVAE groups by Mann–Whitney *U*-Test. Univariable and multivariable Cox proportional hazard regression analysis were used to identify factors predictive of CVAE and OS. Clinical factors (age, sex, race, BMI, smoking status, performance status, pre-existing heart disease, clinical disease stage, tumor size, location and histology, receipt of chemotherapy, and radiation dose and modality) and radiation mean dose to heart were assessed for potential association with CVAE and survival. Risk factors were selected for OS multivariable model in backward stepwise manner using a threshold of removal of *P* > 0.2; and selected for the CVAE multivariable model if *p* < 0.1 in univariable analysis or clinically relevant to CVAE. A two-sided *p*-value of < 0.05 was considered statistically significant. All statistical analyses were performed using IBM SPSS Statistics for Windows, Version 26.0. Armonk, NY: IBM Corp.

## Results

### Patient population

A total of 193 participants with NSCLC were included in the study. Mean age was 64 ± 9 years, 45% were female and 91% were Caucasian. Mean BMI was 28 ± 6 kg/m^2^. Out of 193 patients, 89 (46%) were diagnosed with hypertension, 45 (23%) with dyslipidemia, 32 (17%) with diabetes mellitus, while 93% were tobacco product users [former (71%) or active (22%)]. Pre-existing CV disease was present in 22% of patients, out of which 32 (17%) had coronary artery disease, 7 (4%) cardiomyopathy or heart failure, 6 (3%) non-coronary atherosclerotic vascular disease, and 3 (2%) arrhythmias. Adenocarcinoma was diagnosed in 49% of patients while squamous cell carcinoma in 36%. Most patients had NSCLC stages IIIA (41%) and IIIB (40%), while 8% had stage II and 4% stage IV. Recurrent NSCLC was present in 6% of patients. Photon-radiation was administered in 59% and proton therapy in 40% of the patients. The range of radiation dose in this population was 60–75 Gy (median 74 Gy). Most patients in this cohort (59%) received ≥74 Gy of radiation, while 33% received 66 to 73 Gy and 8% received <66 Gy. Mean heart dose ± SD in this cohort was 14 ± 10 Gy (Table 1).

**TABLE 1:** Demographic and baseline clinical characteristics of 193 patients with NSCLC treated with concurrent chemoradiation categorized based on their baseline coronary calcium scores.

Characteristics	Total (*n* = 193)	CAC = 0 (*n* = 51)	CAC >0 (*n* = 142)	*P*-value	0< CAC <100 (*n* = 48)	CAC ≥100 (*n* = 94)	*P*-value
Age, mean (SD), years	64 (9)	57 (10)	66 (8)	**<0.0001**	64 (9)	68 (7)	**0.002**
Male sex, *N* (%)	106 (55)	18 (35)	88 (62)	**0.001**	22 (46)	66 (70)	**0.006**
Race, *N* (%)				0.385			0.247
- White	176 (91)	45 (88)	131 (92)		43 (90)	88 (94)	
- Other	17 (9)	6 (12)	11 (8)		5 (10)	6 (6)	
BMI, mean (SD), kg/m^2^	28 (6)	28 (6)	28 (5)	0.572	28 (4)	29 (6)	0.631
Smoking status, *N* (%)				0.218			0.036
- Never	14 (7)	6 (12)	8 (6)		6 (13)	2 (2)	
- Previous	137 (71)	32 (63)	105 (74)		34 (71)	71 (76)	
- Active	42 (22)	13 (25)	29 (20)		8 (17)	21 (22)	
Hypertension, *N* (%)	89 (46)	22 (43)	67 (47)	0.619	18 (38)	49 (52)	0.099
Dyslipidemia, *N* (%)	45 (23)	7 (14)	38 (27)	0.059	10 (21)	28 (30)	0.254
Diabetes mellitus, *N* (%)	32 (17)	5 (10)	27 (19)	0.249	4 (8)	23 (25)	**0.019**
Pre-existing cardiovascular disease, *N* (%)	42 (22)	1 (2)	41 (29)	**<0.0001**	1 (2)	40 (43)	**<0.0001**
Tumor histology, *N* (%)				0.076			**0.015**
- Adenocarcinoma	95 (49)	32 (63)	63 (44)		21 (44)	42 (45)	
- SCC	70 (36)	13 (25)	57 (40)		14 (29)	43 (46)	
- Other	28 (15)	6 (12)	22 (16)		13 (27)	9 (10)	
Disease stage, *N* (%)				**0.010**			0.090
- II	16 (8)	2 (4)	14 (10)		1 (2)	13 (14)	
- IIIA	79 (41)	12 (23)	67 (47)		23 (48)	44 (47)	
- IIIB	78 (40)	29 (57)	49 (35)		19 (40)	30 (32)	
- IV	8 (4)	3 (6)	5 (4)		3 (6)	2 (2)	
Recurrent disease	12 (6)	5 (10)	7 (5)		2 (4)	5 (5)	
Gross tumor volume, mean (SD), cm^3^	129 (134)	126 (166)	130 (122)	0.849	121 (119)	134 (124)	0.562
Radiation dose, *N* (%)				0.070			0.685
- <66 Gy	16 (8)	5 (10)	11 (8)		5 (10)	6 (6)	
- 66–73 Gy	64 (33)	23 (45)	41 (29)		13 (27)	28 (30)	
- ≥74 Gy	113 (59)	23 (45)	90 (63)		30 (63)	60 (64)	
Mean heart dose (SD), Gy	14 (10)	16 (10)	14 (9)	0.284	15 (9)	13 (9)	0.376
Radiation technique, *N* (%)				0.567			1.000
- Photon	114 (59)	32 (63)	82 (58)		28 (58)	54 (58)	
- Proton	78 (40)	29 (37)	59 (42)		20 (42)	39 (42)	

SD, standard deviation; BMI, body mass index; SCC, squamous cell carcinoma; CAC, coronary artery calcium scoring. Bold numbers indicate a statistically significant *p*-value of <0.05.

### Coronary artery calcification assessment

A total of 74% of the patients in our cohort (142/193) had a CAC more than 0 Agatston units (AU), 49% had CAC ≥100 AU and 36% CAC ≥300 AU. Median CAC was 78 AU (IQR: 0, 761.9), Demographic and clinical characteristics of the patients with 0 AU, >0 AU and ≥100 AU are presented in Table 1. Compared to the patients with CAC of 0 AU, patients with CAC >0 AU were older (mean age 57 ± 10 years vs. 66 ± 8 years, *p* < 0.001), more likely to be male (35% vs. 62%, *p* = 0.001), have pre-existing CV disease (2% vs. 29%, *p* < 0.0001) and be diagnosed at earlier stages of NSCLC (Stage II and IIIA in 4 and 23% vs. 10 and 47%, respectively; *p* = 0.01) (Table 1). Among patients with CAC >0 AU, higher CAC score values (CAC ≥100 AU vs. <100 AU) were noted in older patients (68 ± 7 years vs. 64 ± 9 years, *p* = 0.002), men (70% vs. 46%, *p* < 0.006), former or active smokers (98% vs. 88%, *p* = 0.036), patients with diabetes (25% vs. 8%, *p* = 0.019) pre-existing CV disease (43% vs. 2%, *p* < 0.0001) and patients with SCC over other NSCLC types (46% vs. 29%, *p* = 0.015) (Table 1).

### Cardiac events

**FIGURE 1:**
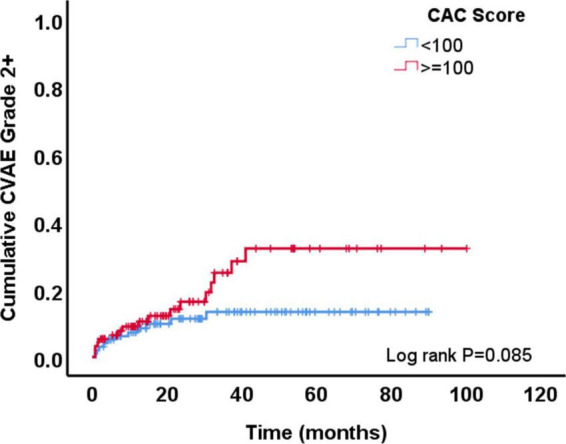
Development of grade ≥2 cardiovascular adverse events (CVAE) overtime among patients with coronary artery calcium (CAC) score <100 AU vs. those with a score ≥100 AU.

**FIGURE 2:**
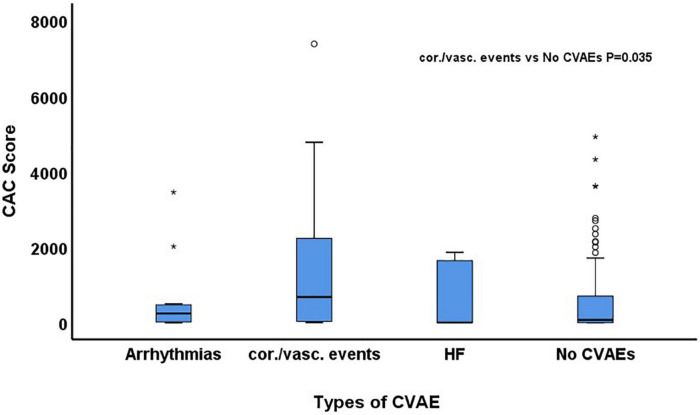
Comparison of baseline coronary artery calcium (CAC) score among patients who developed arrhythmias, coronary/vascular (cor./vasc.) events and heart failure (HF) and those who did not develop any cardiovascular adverse event (CVAE).

During a median follow-up of 24.3 months (IQR: 10.9–51.7), 29 patients (15%) developed at least one grade ≥2 CVAE. Two patients (1%) had a grade 2 event, 21 (11%) a grade 3 event, 4 (2%) a grade 4 event, and 2 (1%) a grade 5 event. Out of the 29 patients who developed a grade ≥2 CVAE, 11 (38%) developed a coronary/vascular event, 12 (41%) atrial fibrillation, and 6 (21%) heart failure. In the univariable Cox regression analysis, CVAEs of grade ≥2 were associated with higher CAC score [HR 1.04 (per 100 AU), 95% CI 1.01–1.06, *p* = 0.003], pre-existing heart disease (HR 2.17, 95% CI 1.01–4.67, *p* = 0.048), and specifically history of heart failure (HR 5.81, 95% CI 1.68–20.11, *p* = 0.005) (Table 2). In the Cox regression analysis, CAC score [HR: 1.04 (per 100 AU), 95% CI: 1.01–1.08, *p* = 0.022] and prior history of heart failure (HR: 4.76, 95% CI: 1.29–17.50, *p* = 0.019) remained significantly and independently associated with the development of grade ≥2 CVAEs (Table 2 and Figure 1). When patients with a CAC score ≥100 AU were compared to those with a CAC score <100 AU, the former had a higher incidence of grade ≥2 CVAE with a trend toward statistical significance (18% vs. 11%, *p* = 0.085; Figure 1). In a sub-analysis evaluating the type of the CVAE, it was the coronary/vascular events that were significantly associated with higher baseline CAC (median: 676 AU vs. 73 AU, *p* = 0.035; Figure 2).

**TABLE 2:** Association of demographic and baseline clinical characteristics with development of grade ≥2 CVAEs based on univariable and multivariable Cox regression analysis.

Variables	Univariable analysis			Multivariable analysis		
		
	HR	95% CI	*P*-value	HR	95% CI	*P*-value
CAC score, 100 AU	1.04	1.01–1.06	**0.003**	1.04	1.01–1.08	**0.022**
CAC score (<100 AU vs. >=100 AU)	0.55	0.26–1.16	0.118			
Age, years	1.03	0.99–1.07	0.183	1.00	0.95–1.05	0.957
Gender (female vs. male)	0.67	0.31–1.41	0.292			
Race (white vs. other)	2.86	0.38–20.0	0.308			
BMI	1.01	0.94–1.08	0.808			
Smoking status						
–Never vs. current	1.73	0.41–7.25	0.455			
–Previous vs. current	1.32	0.50–3.52	0.576			
Pre-existing heart diseases						
–CAD vs. none	1.90	0.76–4.77	0.170	0.71	0.21–2.48	0.600
–PAD/TIA/AAA vs. none	1.09	0.15–8.16	0.935	0.67	0.08–5.59	0.720
–Heart failure vs. none	5.81	1.68–20.11	**0.005**	4.76	1.29–17.50	**0.019**
Tumor histology						
–Adenocarcinoma vs. SCC	0.72	0.31–1.67	0.451			
–Others vs. SCC	1.35	0.51–3.57	0.542			
Stage						
–IIIA vs. II	0.41	0.15–1.07	0.067	7.49	0.81–69.53	0.077
–IIIB vs. II	0.30	0.11–0.84	**0.022**	3.77	0.42–33.57	0.235
–IV vs. II	–	–	0.970	3.67	0.39–34–64	0.256
–Recurrence vs. II	0.17	0.02–1.38	0.096			
Gross tumor volume, cm^3^	1.000	0.996–1.00	0.571			
Tumor location (left/mediastinum vs. right)	1.060	0.50–2.24	0.873	1.12	0.50–2.55	0.781
Mean heart dose, Gy	0.99	0.95–1.03	0.447	1.00	0.96–1.05	0.954
KPS (≥80 vs. <80)	0.62	0.23–1.62	0.325			
Total radiation dose, Gy	1.06	0.96–1.16	0.263			
Radiation technique (proton vs. photon)	0.92	0.44–1.93	0.827			

BMI, body mass index; SCC, squamous cell carcinoma; CAC, coronary artery calcium scoring; KPS, Karnofsky performance status; HR, hazard ratio; CI, confidence interval. Bold numbers indicate a statistically significant *p*-value of <0.05.

### Overall survival

**FIGURE 3:**
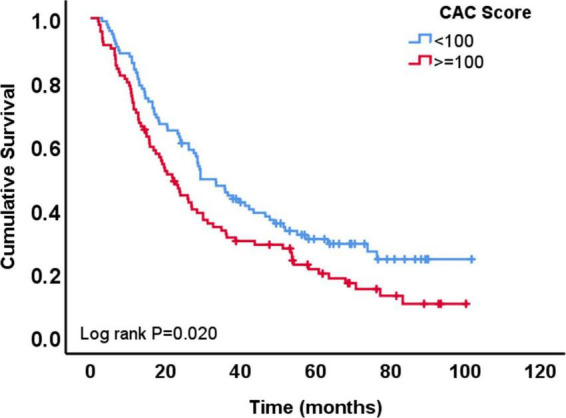
Overall survival of patients with coronary artery calcium (CAC) score <100 AU vs. those with ≥100 AU.

A total of 148 patients (77%) died during the follow-up period of the study. The median survival time was 27 months after chemoradiation therapy in the total cohort. Patients with low CAC score (<100 AU) had longer survival time (29 months) compared to patients with high CAC score (>=100 AU; 22 months; *p* = 0.051). Univariable Cox regression analysis showed that OS was longer among patients with lower CAC score (HR 0.68, 95% CI 0.49–0.94, *p* = 0.021 for CAC <100 AU vs. ≥100 AU), female sex, patients with adenocarcinoma vs. SCC, patients with lower gross tumor volume, higher Karnofsky performance status and those who received lower dose of radiation (Table 3). In the multivariable Cox regression analysis, lower CAC remained independently associated with longer OS (CAC <100 vs. ≥100 AU, HR: 0.61, 95% CI: 0.41–0.90, *p* = 0.013) (Table 3 and Figure 3).

**TABLE 3:** Association of demographic and baseline clinical characteristics with overall survival based on univariable and multivariable Cox regression analysis.

Variables	Univariable analysis			Multivariable analysis		
		
	HR	95% CI	*P*-value	HR	95% CI	*P*-value
CAC score, 100 AU	1.01	0.99–1.02	0.278			
CAC Score (<100 AU vs. ≥100 AU)	0.68	0.50–0.94	**0.021**	0.61	0.41–0.90	**0.013**
Age, years	1.02	0.99–1.03	0.179			
Gender (female vs. male)	0.65	0.47–0.90	**0.010**	0.64	0.45–0.91	**0.012**
Race (white vs. other)	0.98	0.56–1.72	0.946			
BMI	1.00	0.97–1.03	0.864			
Smoking status:						
–Never	0.83	0.41–1.69	0.610			
–Previous	1.06	0.72–1.58	0.757			
–Current	1.00					
Pre-existing heart disease:						
–CAD vs. none	1.09	0.69–1.71	0.727	0.62	0.36–1.05	0.076
–PAD/TIA/AAA vs. none	0.89	0.36–2.19	0.806	0.42	0.16–1.11	0.082
–Heart failure vs. none	1.97	0.92–4.24	0.083	1.38	0.60–3.21	0.449
Tumor histology:						
–SCC	1.00					
–Adenocarcinoma	0.66	0.47–0.93	**0.020**	0.75	0.43–1.30	0.313
–Other	0.60	0.36–1.00	**0.048**	0.58	0.33–1.00	0.050
Stage						
–II	1.00					
–IIIA	1.03	0.55–1.92	0.921			
–IIIB	1.18	0.63–2.18	0.608			
–IV	1.02	0.38–2.73	0.964			
–Recurrence	0.73	0.30–1.78	0.486			
Gross tumor volume, cm^3^	1.001	1.000–1.002	**0.006**			
Tumor location (left/mediastinum vs. right)	0.860	0.62–1.19	0.355			
Mean lung dose, Gy	1.05	1.001–1.095	**0.046**	1.04	0.99–1.09	0.144
Mean heart dose, Gy	1.00	0.98–1.02	0.843			
KPS (≥80 vs. <80)	0.63	0.41–0.99	**0.043**	0.62	0.37–1.02	0.060
Total radiation dose, Gy	0.96	0.93–0.99	**0.012**	0.95	0.91–0.99	**0.016**
Radiation technique (proton vs. photon)	1.19	0.86–1.66	0.284			

BMI, body mass index SCC, squamous cell carcinoma CAC, coronary artery calcium scoring; KPS, Karnofsky performance status; HR, hazard ratio; CI, confidence interval. Bold numbers indicate a statistically significant *p*-value of <0.05.

## Discussion

This study evaluated the role of CAC on standard of care, oncologic NCCT in predicting CVAEs in patients with NSCLC who are treated with concurrent chemoradiation therapy. Our findings suggest that even in the era of contemporary RT techniques reducing radiation exposure to the heart, CVAEs (grade ≥2) remain frequent among patients with NSCLC, being observed in 1 out of 7 patients in our cohort over a median follow-up of 24 months. CAC on standard of care, oncologic NCCT predicts the development of CVAEs, specifically coronary/vascular events, and OS, independent of traditional CV risk factors, total radiation dose, mean heart dose, and radiation modality.

The incidence of CVAEs in patients with NSCLC has been reported to be in the range of 10%. Dess et al. studied 125 patients with locally advanced NSCLC enrolled in four prospective RT trials at two centers in Michigan from 2004 to 2013 (3). They reported a 24-month cumulative incidence of grade ≥3 CVAEs of 11% (3). This is in line with the findings of this study, where the incidence of grade 3 or higher CVAEs was 14% among patients with NSCLC treated between 2009 and 2014, suggesting that RT-related CVAEs remain prevalent.

Coronary artery calcification scoring on ECG-gated, non-contrast, prospective cardiac CT imaging has been shown to predict major CV outcomes beyond traditional CV risk factors in the general population and has been proposed by expert guidelines as an approach to identify individuals with intermediate CV risk who will benefit from statin therapy (21). However, patients with cancer who are actively treated with chemoradiation are frequently overwhelmed with the burden of tests they have to complete and adding an imaging study, which may not directly affect their cancer treatment, may be challenging. However, recent expert recommendations support the qualitative or quantitative evaluation of NCCT for CAC (22). CAC measured on non-gated CT correlated well with the traditional gated CAC protocol acquired by a multi-detector CT (MDCT) in a study of 163 healthy participants who underwent both imaging studies on the same day (Spearman correlation coefficient 0.83, *P* < 0.001) (15). In a recent retrospective analysis of 428 patients with locally advanced lung cancer, a deep learning method was used to generate an Agatston-like CAC score and showed that CAC ≥1 was associated with increased risk mortality and a trend toward increased risk of major adverse cardiac events (23). Our study included patients with NSCLC stage II–IV treated with concurrent chemoradiation therapy, including photon or proton RT. A detailed characterization of the patients and their treatment was available in our study and used for our analysis. Furthermore, CAC scores were calculated as opposed to a qualitative description of presence or absence of coronary calcifications. Our findings confirmed that CAC on NCCT is an independent predictor of mortality. Furthermore, our study showed that CAC on NCCT independently predicts adverse atherosclerotic CV events in patients with NSCLC.

The strengths of our study include the thorough characterization of patients with NSCLC including details about their RT, the quantitative analysis of CAC as opposed to qualitative analysis and the long follow-up period (2 years). Despite its strengths, our study also has limitations. The small sample size and the retrospective nature of the study with its inherent risk for bias are two main ones. Multivariable regression analysis was performed as a means of addressing the risk of bias. Additionally, CAC when analyzed as a continuous variable was significantly associated with grade ≥2 CVAE but not as a categorical variable (<100 vs. ≥100 AU). In contrary, CAC as categorical variable (<100 vs. ≥100 AU) was significantly associated with OS but not as a continuous variable. This discrepancy is likely related to the small sample size of the study and our findings need to be confirmed in larger cohorts. Finally, even though we reported the prevalence of baseline CV risk factors in the patients of our cohort, we did not have sufficient data to calculate their predicted 10-year atherosclerotic risk and evaluate how CAC performed in each risk category.

In conclusion, CVAEs are frequently observed in patients with NSCLC treated with concurrent chemoradiation therapy. CAC on standard of care, oncologic NCCT independently predicts the development of coronary/vascular events and worse OS. We propose the use of CAC on NCCT scans for the routine assessment of the CV risk in patients with NSCLC which will allow for better risk stratification and implementation of treatment strategies that will mitigate CVAEs.

## Data availability statement

The original contributions presented in this study are included in the article/supplementary material, further inquiries can be directed to the corresponding author.

## Ethics statement

The studies involving human participants were reviewed and approved by The University of Texas MD Anderson Cancer Center Institutional Review Board. The patients/participants provided their written informed consent to participate in this study.

## Author contributions

EK and TX collected the data, performed the initial statistical analysis, and drafted the manuscript. EK adjudicated the adverse cardiovascular events. JL-M and YL performed the coronary artery calcium scoring analysis, and edited the manuscript. JI-C helped with data collection. AD and ZL overlooked the study and provided guidance with the data collection, analysis, and manuscript writing. RL assisted with the data analysis. All authors have contributed to the interpretation of data analysis and editing of the manuscript, and reviewed the final version of the manuscript.
